# m^7^G regulator-mediated methylation modification patterns define immune cell infiltration and patient survival

**DOI:** 10.3389/fimmu.2022.1022720

**Published:** 2022-10-28

**Authors:** Lu Wang, Xing Hu, Xiaoni Liu, Yingmei Feng, Yuan Zhang, Jing Han, Xuqing Liu, Fankun Meng

**Affiliations:** ^1^ Ultrasound and Functional Diagnostic Center, Beijing Youan Hospital, Capital Medical University, Beijing, China; ^2^ Beijing Institute of Hepatology, Beijing Youan Hospital, Capital Medical University, Beijing, China; ^3^ Department of Science and Technology, Beijing Youan Hospital, Capital Medical University, Beijing, China

**Keywords:** hepatocellular carcinoma, m^7^G modification, tumor microenvironment, immunotherapy, prognosis, drug response

## Abstract

Numerous studies have demonstrated the important roles of epigenetic modifications in tumorigenesis, progression and prognosis. However, in hepatocellular carcinoma, the potential link between N^7^-methylguanosine (m^7^G) modification and molecular heterogeneity and tumor microenvironment (TME) remains unclear.

**Method:**

We performed a comprehensive evaluation of m^7^G modification patterns in 816 hepatocellular carcinoma samples based on 24 m^7^G regulatory factors, identified different m^7^G modification patterns, and made a systematic correlation of these modification patterns with the infiltration characteristics of immunocytes. Then, we built and validated a scoring tool called m^7^G score.

**Results:**

In this study, we revealed the presence of three distinct m^7^G modification patterns in liver cancer, with remarkable differences in the immunocyte infiltration characteristics of these three subtypes. The m^7^G scoring system of this study could assess m^7^G modification patterns in individual hepatocellular carcinoma patients, could predict TME infiltration characteristics, genetic variants and patient prognosis. We also found that the m^7^G scoring system may be useful in guiding patients’ clinical use of medications.

**Conclusions:**

This study revealed that m^7^G methylation modifications exerted a significant role in formation of TME in hepatocellular carcinoma. Assessing the m^7^G modification patterns of single patients would help enhance our perception of TME infiltration characteristics and give significant insights into immunotherapy efficacy.

## Introduction

Liver cancer is one of the most common malignancies worldwide, and the primary pathological type of the disease is hepatocellular carcinoma (HCC). The incidence of liver cancer is expected to exceed 1 million cases by 2025 (1). Liver cancer is the fourth leading cause of cancer-related deaths worldwide (2). Liver cancer has become one of the most challenging problems in the world due to its high incidence and mortality rate. Currently, the mainstay clinical treatments for liver cancer involve surgical resection, liver transplantation, ablation and interventional embolization therapy, etc. These traditional treatments have been made remarkable progress and been successful in the treatment of early stage liver cancer. However, as liver cancer is characterized by high invasiveness, metastasis and recurrence rate, and most of the liver cancers are in advanced stages when diagnosed, the treatment of middle and late stage liver cancer has long been a clinical challenge, and the clinical outcome of patients is still not satisfactory (3). Over the past few years, there have been numerous studies devoted to treat liver cancer with targeted drugs and immunotherapy, and significant progress has been made with molecularly targeted therapeutic agents represented by the multikinase inhibitor sorafenib. However, the 5-year survival rate of sorafenib for HCC is very poor, and the prognosis for advanced hepatocellular carcinoma remains poor with limited successful cases (4). Immunotherapy has shown impressive clinical efficacy in a small group of patients, while the majority of patients, unfortunately, have received little or no clinical benefits, falling far behind of their clinical needs (5, 6). Thus, it is urgently needed to further develop diagnostic markers and therapeutic targets in hepatocellular carcinoma. In order to develop more effective therapeutic strategies to improve the patients’ clinical prognosis, a deeper comprehension of the molecular mechanisms underlying hepatocarcinogenesis and malignant progression becomes even more essential.

During the development of chronic liver disease and cirrhosis, hepatocytes gradually accumulate a large number of genetic mutations and epigenetic changes (7), becoming a major pathogenetic basis of hepatocellular carcinoma. Numerous studies have demonstrated that epigenetic modifications play significant roles in tumorigenesis, progression, treatment, and prognosis (8, 9). RNA methylation modifications are widely present in life processes as the third level of epigenetics, and over 150 RNA modifications have been identified. The main RNA methylation modifications widely found on mammalian genes include m6A, m1A and m^7^G, etc (10). RNA methylation is the occurrence of methylation modifications at different locations on the RNA molecule, and these methylation modifications could regulate processes such as RNA variable splicing (11), exonucleation (12), stabilization (13), translation (14), and immunogenicity (15). When methylation modification occurs at the nitrogen atom at position 7 of the guanine of the RNA, it is described as N^7^-methylguanosine (m^7^G). In the process of post-transcriptional regulation, m^7^G modification is one of the commonest base modifications (16). Under normal conditions, m^7^G methylation modifications within human mRNA are basically concentrated in the 5’ untranslated region and in environments rich in both A and G bases, while m^7^G modifications improve the stability of mRNA (17, 18). The m^7^G methylation modification is a dynamic biological process that allows the organism to adapt to a constantly changing environment. For example, m^7^G can be dynamically regulated by H2O2 and heat shock treatment and then evolves to become abundant in the coding region and 3’UTR region of genes. Besides being found on mRNAs, m^7^G methylation modifications are present in tRNAs, miRNAs, and rRNAs (19–21).

In recent years, m^7^G methylation has become a rising hotspot in RNA modification research. With increased research, m^7^G modifications have been demonstrated to play significant roles in regulation of normal human biological processes (16, 22). And abnormal m^7^G methylation modifications have also been found to be closely associated with dysregulation of RNA, which may ultimately lead to disease and cancer (23). Several studies have detected the presence of m^7^G modifications in HCC, and m^7^G modifications were closely associated with the development of HCC (24–26). Chen et al. found that m^7^G tRNA modification and its catalase metttl1 were expressed elevated in HCC, and METTL1-mediated m^7^G tRNA modification could promote mRNA translation, which was shown to accelerate the development and progression of hepatocellular carcinoma and correlated with poor prognosis of hepatocellular carcinoma by *in vitro* and *in vivo* experiments (24). Xia et al. found that the RNA methyltransferase *WDR4* was highly expressed in hepatocellular carcinoma, and upregulation of *WDR4* expression enhanced the methylation level of m^7^G in hepatocellular carcinoma. *WDR4* promotes tumor cells metastasis and resistance to sorafenib *via* epithelial-mesenchymal transition (EMT), thereby promoting the proliferation of hepatocellular carcinoma cells (27).

Tumorigenesis and progression is a multistep process which involves not only epigenetic variation in tumor cell, but also the tumor microenvironment (TME) plays essential roles in tumor development. The hepatocellular carcinoma tumor microenvironment is a dynamical system composed of cancer cell, cytokine, extracellular matrix, and immunocyte subpopulations (28), and the interactions between hepatocellular carcinoma cells and various immune components in the TME are diverse and complex. Almost all major tumor immune cells are important in TME of hepatocellular carcinoma. TME is tightly associated with the initiation, progression and metastasis stages of HCC (29). Recently, several studies have revealed that RNA methylation impacts the effectiveness of tumor therapy by modulating tumor immunity in addition to directly influencing tumor development (30–32). Nevertheless, it is unclear whether m^7^G methylation modifications in hepatocellular carcinoma also play a potential role in tumor microenvironment (TME) formation. A comprehensive evaluation of the variability and sophistication of TME landscape may help enhance the guidance and prediction of immunotherapeutic responses and would help to identify new therapeutic targets.

In this study, we comprehensively assessed the association of m^7^G modification patterns and immunocytes infiltration features by analyzing genomic information from total 816 HCC samples. We identified three m^7^G modification patterns with unsupervised clustering, and also found that the TME cell infiltration characteristics among these three subtypes were distinctly different. Moreover, with the consideration of the heterogeneity of m^7^G modification among individual patients, we formulated a score system to quantitate m^7^G modification patterns in individual patients and to predict the clinical response of patients to immune checkpoint inhibitor (ICI) therapy.

## Materials and methods

### Patient and clinical samples

A total of 10 pairs of HCC and adjacent non-cancerous tissues were collected, all from Beijing Youan Hospital. The samples were examined by three experienced pathologists.All patients provided informed consent,and the study protocol was approved by the Ethics Committee of Beijing Youan Hospital.

### Immunohistochemistry

Immunohistochemical (IHC) staining was performed with NUDT16 antibody (12889-1-AP, proteintech). Immunohistochemical examination was performed by two pathologists, and positive staining cells were found to be visible in tumor and paired adjacent tissues. The IHC score was computed by the percentage of stained cells and the intensity of staining (33).

### Collecting and pre-processing data

**Figure 1:**
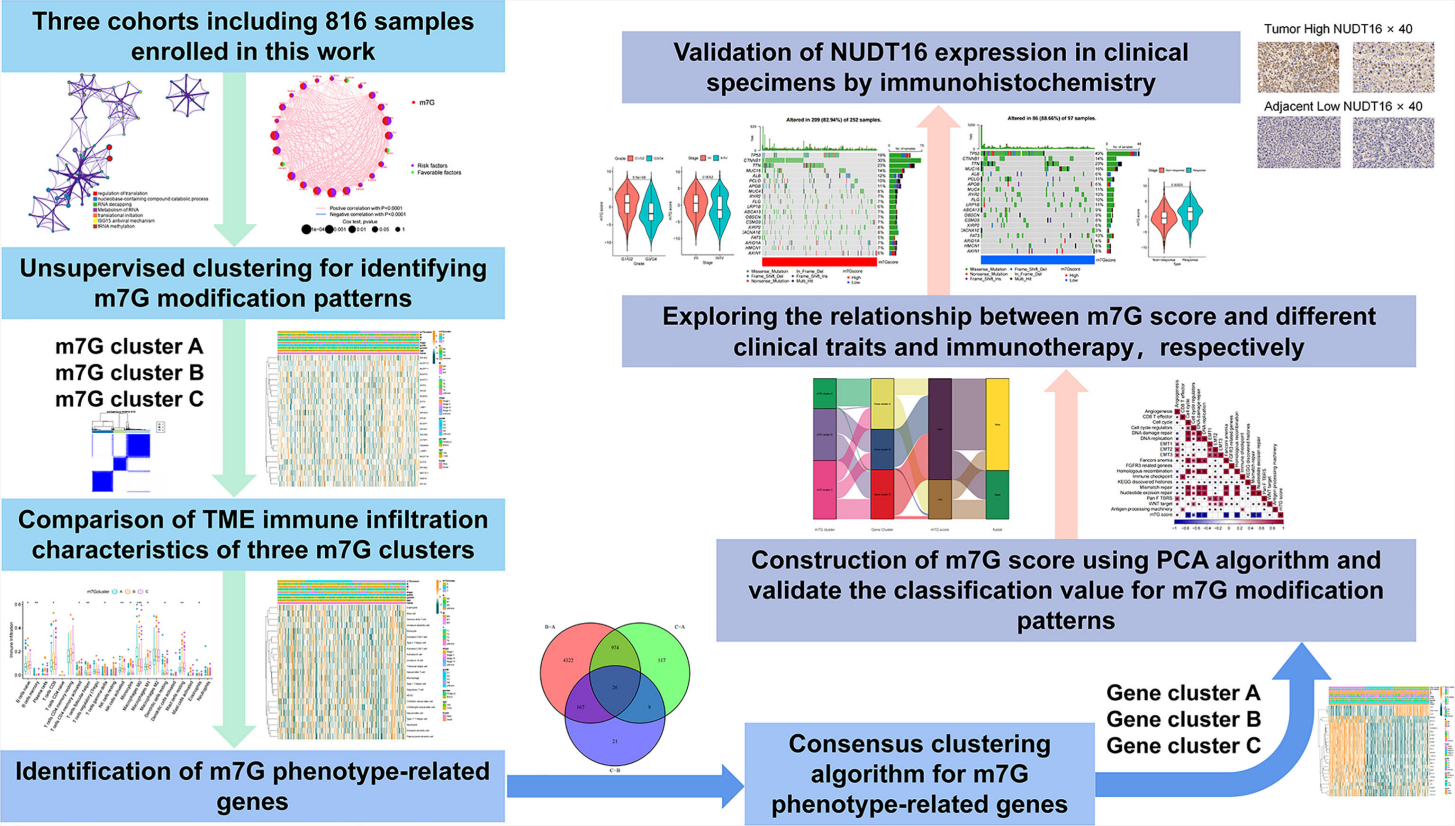
Overview of this work.

Our study workflow was illustrated in **Figure 1**. We downloaded RNA expression data and clinical information of HCC samples from TCGA database, GEO database and ICGC database, and RNA expression data of 50 normal liver tissue cases from TCGA database. Finally, a total of 364 tumor cases from TCGA-LIHC cohort in TCGA database, 231 tumor cases from LIRI-JP cohort in ICGC database and 221 tumor cases from GSE14520 cohort in GEO database were included. These tumor samples all have complete clinical information including survival time and status, and all have more than half of the gene expression values. For the TCGA-LIHC cohort, the downloaded FPKM values were converted into log2 (TPM+1) values. Somatic mutation data were obtained from TCGA database. The clinical characteristics of the TCGA-LIHC and GSE14520 cohorts were listed in **Table S1** and **Figures S1A, B**. We used R (version 4. 1. 2) to analyze the data.

### Identification of molecular subgroups and calculating DEGs

Through previous studies, we extracted 24 genes related to m^7^G. Differential expression of these 24 genes between 50 normal and 364 tumour cases from TCGA datebase was analyzed by the”limma” package in R. We extracted the expression matrix of these genes in the tumor samples in TCGA-LIHC cohort, and consensus clustering was performed using “ConsensusClusterPlus” package (34). DEGs between the three clusters were analyzed using ‘limma’ package with the cutoff criteria of |log2 fold change (FC)| > 1 and P-value< 0. 05.

### Functional analyses, TIME evaluation and immunophenoscore

To analyze the functions of the shared DEGs among the above three subtypes, Gene Ontology Function was obtained using the the”clusterprofiler”package in R. We obtained the a gene set of “h.all.v7.5.1. symbols” from MSigDB. Mariathasan et al. built gene sets in which genes related to certain biological processes are stored (35). Based on the above gene set, GSVA was performed using the “GSVA” and “limma” packages to visualize alterations in signaling pathways among three subtypes (36). We used the ESTIMATE algorithm to calculate the immune score. The immunophenotype (IPS) is a better predictor of response to anti-CTLA4 and anti-PD1 regimens, and we downloaded the relevant scores from The Cancer Immunome Atlas (TCIA) for the TCGA-LIHC cohort.

### Estimation of TME cell infiltration

Cohorts of 23 immune-related cells were downloaded and collated (37). 23 immune infiltrating cells in tumor samples were analyzed by ssGSEA enrichment using the “GSVA” software package. The score calculated using ssGSEA were performed to express relative abundance of different immunocytes in the samples (38).

### m^7^G score construction

In order to quantitate the m^7^G modification pattern of individual tumors, we constructed a system called m^7^G score, which was established as follows:

We selected overlapping DEGs found in different m^7^G clusters. Prognostic analysis was then performed by using univariate Cox regression analysis for the above DEGs. Extraction of genes with significant prognosis was used to construct the m^7^G score by principal component analysis (PCA). We extracted PC1 and PC2 as signature scores. We then used a similar approach to define the m^7^G score as in previous studies (39, 40). m^7^Gscore=∑(PC1i+PC2i), i is the expression of genes associated with the m^7^G phenotype.

### Collection of immune checkpoint blockade clinical information and gene expression

The result of a systematic search we performed was in the inclusion of an immunotherapy cohort: uroepithelial carcinoma intervening with atezolizumab (IMvigor210 cohort). The downloaded data were normalized and converted to TPM values.

### Drug sensitivity analysis

We utilized the “pRRophetic” package to analyze the sensitivity to different m^7^G clusters to different small molecule drugs. We utilized CellMiner database to assess the association between different m^7^G regulators and drug sensitivity (41).

### Cell culture and transfection

Human liver cancer cell line (Huh-7 and HepG2) was purchased from the American Type Culture Collection. Huh-7 cells and HepG2 cells were cultured in DMEM (Gibco) containing 10% fetal bovine serum (Gibco) and were cultured in a humidified incubator with 5% CO2 at 37°C. Si-NUDT16 (Suzhou, China, sequences-1: 5’GGUUAAUAAUAGAGAGCUAUG’3;sequences-2:5’CGACAGAUGUUGAGGAGAAUG’3) were used for transfection.

### Cell adhesion assay

Add 50 µl of fibronectin (Biocoat) or vitronectin (PeproTech) to a 96-well plate (Corning) and incubate overnight at 4 degrees Celsius. The unbound fibronectin was washed off the next day and the 96-well plate was closed with 1% BSA for two hours. 10,000 cells were added to each well and cultured in DMEM (Gibco) for 2 hours. Unbound cells were washed away, fixed with 4% paraformaldehyde for 15 min and stained with 0.1% crystal violet for 15 min.

### Statistical analyses

The Student’s t-test was applied to normally distributed variables and the Wilcoxon rank sum test was applied to non-normally distributed variables. The Kruskal-Wallis test and one-way ANOVA were applied for the nonparametric and parametric methods, respectively (42). Survival curves were prognostically analyzed using the Kaplan-Meier method, and log-rank tests were used to determine the significance of differences. We used a univariate Cox regression model to calculate the hazard ratios (HR) of m^7^G regulators and m^7^G phenotype-associated genes. Independent prognostic factors were identified by multivariate Cox regression models. We stratified the samples into m^7^G score high and low subgroups using the surv-cutpoint function in the ‘survival’ package. The specificity and sensitivity of m^7^G score were assessed using receiver operating characteristic (ROC) curves, and the area under the curve (AUC) was calculated using the ‘timeROC’ package. The waterfall function of maftools package was used to present the mutation landscape. We used the ‘RCircos’ package to map the copy number variation of 24 m^7^G regulators on 23 pairs of chromosomes. All p-values were bilateral and p-values less than 0. 05 was statistically significant.

## Results

### Landscape of genetic variation of m^7^G regulators in liver cancer

**Figure 2:**
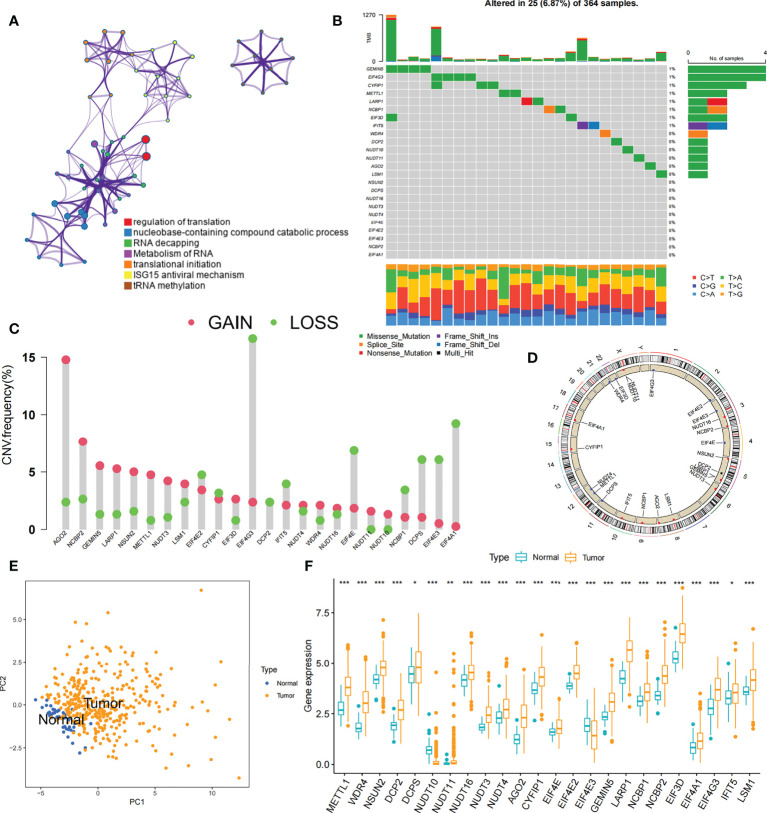
Profiles of genetic alterations of m^7^G regulators in HCC. **(A)** Metascape enrichment network. Clustering annotations were color-coded. **(B)** Among 364 HCC samples, 25 occurred genetic alterations in 24 m^7^G genes with a frequency of 6.87%, consisting mainly of missense mutations. The right-hand side numbers represent the frequency of mutations in individual regulators. Each column indicates a single samples. **(C)** The frequency of CNV mutations in 24 m^7^G regulators was prevalent. The column indicates alteration frequency. The amplification frequency, red dots.; The deletion frequency, green dots. **(D)** CNV of the m^7^G regulator changes position on the chromosome. **(E)** PCA of 24 m^7^G regulators that can distinguish tumor patients from normal patients. **(F)** Differences in mRNA expression levels of 24 m^7^G regulators between HCC and normal patients. Asterisks represent statistical p-values (*P< 0. 05; **P< 0. 01; ***P< 0. 001).

We explored the role of 24 m^7^G RNA methylation-regulated genes of HCC in this study (**Table S2**). GO enrichment and Metascape analysis were performed on 24 m^7^G regulators, and the result showed significantly enriched biological processes (**Figure 2A**; **Figure S1A**). We first established the incidence of somatic mutations in 24 m^7^G regulators in HCC. 25 (6. 9%) samples out of 364 showed genetic alterations in m^7^G regulators, mainly consisting of missense mutations. *GEMIN5* and *EIF4G3* had the highest mutation frequency, followed by *CYFIP1* (**Figure 2B**). Further analysis of the 24 m^7^G regulators showed that CNV mutations were prevalent. *AGO2*, *NCBP2*, *GEMIN5* and *LARP1* all showed extensive CNV amplification. In contrast, *EIF4G3*, *EIF4E*, *DCPS*, *EIF4E3*, and *EIF4A1* had widespread CNV deletions (**Figure 2C**). The CNV alteration positions of the 24 m^7^G regulators on the chromosomes are depicted in **Figure 2D**. Principal component analysis (PCA) was performed on tumor and normal samples, and 24 m^7^G regulators were found to thoroughly separate them (**Figure 2E**). Further analysis yielded that only *NUDT10* and *EIF4E3* were expressively down-regulated in HCC samples, while the other 22 genes were expressively up-regulated in HCC samples (**Figure 2F**). The expression of CNV-amplified m^7^G regulators was markedly higher in HCC specimens than in normal control specimens, such as *AGO2*, *NCBP2*, *GEMIN5* and *LARP1*, while the expression of *EIF4E3* was significantly lower in tumor specimens (**Figures 2C, F**). Furthermore, Spearman correlation analysis was used to evaluate the interregulatory effects between these m^7^G regulators (**Figure S1B**). Cox regression analysis of these m^7^G regulators was performed in relation to the prognosis of HCC patients (**Figures S1C, D**). A forestplot showed that *NUDT16* was considered as a risk factor. The above analyses showed that the genomic and transcriptomic landscapes of m^7^G regulatory factors were linked and different in normal and HCC samples. Therefore, genetic variation and altered expression of m^7^G regulatory factors played significant roles in the development of HCC.

### Identification of m^7^G modification patterns mediated by 24 regulators

**Figure 3:**
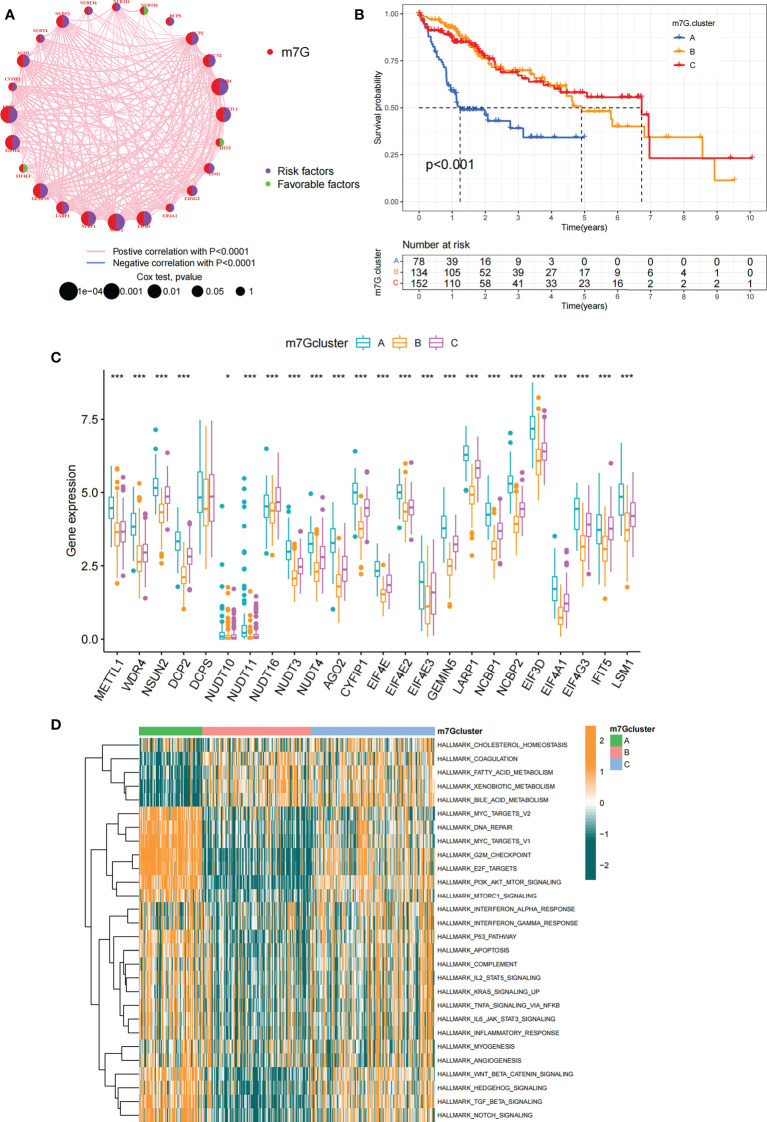
m^7^G modification patterns and related biological pathways. **(A)** Interaction of 24 m^7^G regulators expressed in HCC. The size of every circle indicated the prognostic effects of individual regulators and scaled by p-value. m^7^G regulators, red; favorable factors, green; risk factors, purple. **(B)** Survival curve for survival of 364 HCC patients in TCGA-LIHC cohort of different m^7^G clusters. The numbers of patients in m^7^G cluster A, B, and C are 78, 134, and 152, respectively. **(C)** Comparing the expression of 24 m^7^G regulators between three m^7^G clusters. **(D)** GSVA enrichment analysis displaying the activation status of biological pathways with different m^7^G clusters. The heatmap was made to visualize these biological processes, with yellow representing activated pathways and green representing inhibited pathways. Asterisks represent statistical p-values (*P < 0. 05; ***P < 0. 001).

Data of 364 samples with liver cancer in TCGA-LIHC cohort were used for analysis. In the m^7^G regulator network, the interactions and connections between 24 m^7^G regulators and their prognostic significance in HCC patients were comprehensively described. The results suggested that the intercommunication of these 24 m^7^G regulators may function critically for the formation of distinct m^7^G modification patterns and associated with cancer development and progression (**Figure 3A**). With these assumptions, we used unsupervised clustering to stratify samples into distinct m^7^G modification patterns according to these 24 m^7^G regulators. Accordingly, we stratified three different clusters of modified patterns, including 78 cases in cluster A, 134 cases in cluster B and 152 cases in cluster C (**Table S3**; **Figures S2A–D**). We referred to these subgroups m^7^G cluster A-C, among which m^7^G cluster B and C showed a prominent survival advantage, while m^7^G cluster A had the worst prognosis (**Figure 3B**). Furthermore, we noticed that there were remarkable differences in the expression of m^7^G regulators among distinct m^7^G modification patterns. The vast majority of genotypes were significantly elevated in the m^7^G cluster A subtype, such as *METTL1*, *WDR4*, *DCP2*, etc (**Figure 3C**; **Figure S2E**).

### The m^7^G modification patterns characterized by distinct immune landscapes

**Figure 4:**
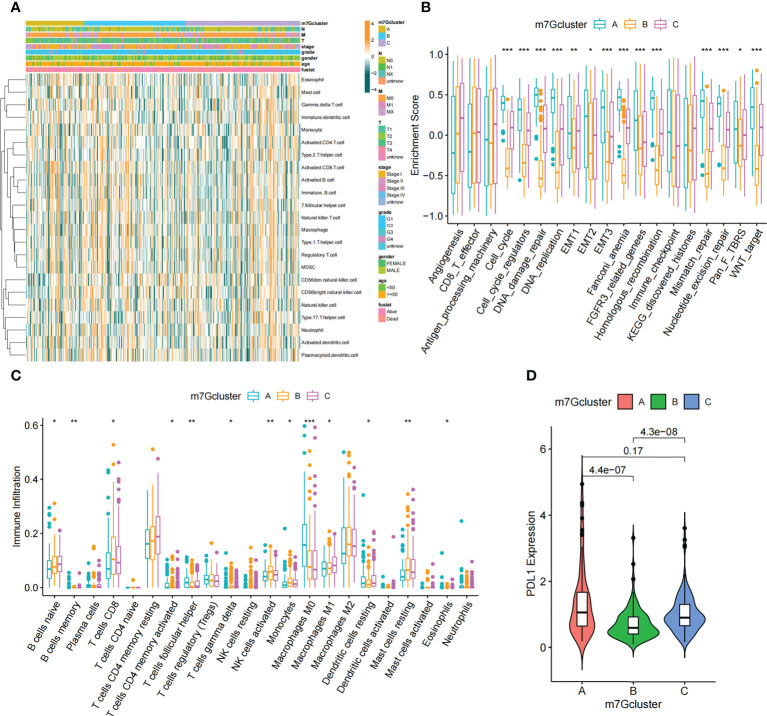
TME characteristics in different m^7^G modification patterns. **(A)** The heatmap used to visualize the infiltration of 23 immunocytes in three m^7^G clusters. Clinicopathological information including age, gender, tumor stage, and m^7^G cluster was shown in the annotation above. Yellow represented high expression of m^7^G regulators and green represented low expression. **(B)** In TCGA-LIHC cohort, m^7^G modification patterns were differentiated by distinct features. (immune-related signature, DNA repair-related signature and matrix-related signature). **(C)** Abundance of TME-infiltrated cells in the three m^7^G clusters. The line in the box indicated the median value and the scatter indicated the abnormal value. The upper and lower ends of the boxes indicated the interquartile range of values. **(D)** Comparison of PD-L1 expression levels of three m^7^G clusters. The whiskers encompassed 1.5 times the interquartile range. The upper and lower ends of the boxes indicated the interquartile range of values. Asterisks represent statistical p-values (*P < 0. 05; **P < 0. 01; ***P < 0. 001).

In order to explore biological behavior behind these distinct m^7^G clusters, GSVA enrichment analysis was performed. The results showed that m^7^G cluster A was remarkably abundant in oncogenic activated related processes, such as Myc targets, PI3K AKT MTOR signaling pathway. While m^7^G cluster B showed enrichment in metabolism-related pathways, such as fatty acid metabolic signaling pathways, as well as m^7^G cluster C that was remarkably abundant in processes related to immune activation, such as inflammatory response and complement signaling pathway (**Figure 3D**). Additionally, we constructed heatmap for visualizing and comparing the relative abundance of 23 immune infiltrating cells under different clusters (**Figure 4A**). Surprisingly to us, subsequent analysis of TME cells infiltrates showed that m^7^G cluster A was abundant in innate immunocyte infiltrates, including activated dendritic cell, immature dendritic cell, plasmacytoid dendritic cell, and MDSC. However, patients in m^7^G cluster A showed no matched survival advantage. Previous studies have found that immune rejection tumors were distinguished by the presence of numerous immunocytes, but these immunocytes remain in the stroma surrounding the tumor cell nests without penetrating the parenchyma of the tumor cell nests. Stromal activation in TME was considered T-cell suppressive (43). GSVA analysis revealed that cluster A was remarkably relevant to stromal activation such as EMT and TGFb, which confirmed our speculations (**Figure 4B**). The above results verified our suspicions. We further characterized the immune infiltration using CIBERSORT and found that antitumor lymphocyte subsets such as CD8 T cell and activated NK cell were mainly abundant in m^7^G clusters B and C (**Figure 4C**). Taking into account that PD-L1 is a proven biomarker to predict immunotherapy response (44), we identified a significant upregulation of PD-L1 expression levels in m^7^G cluster A and C subtypes (**Figure 4D**).

**Figure 5:**
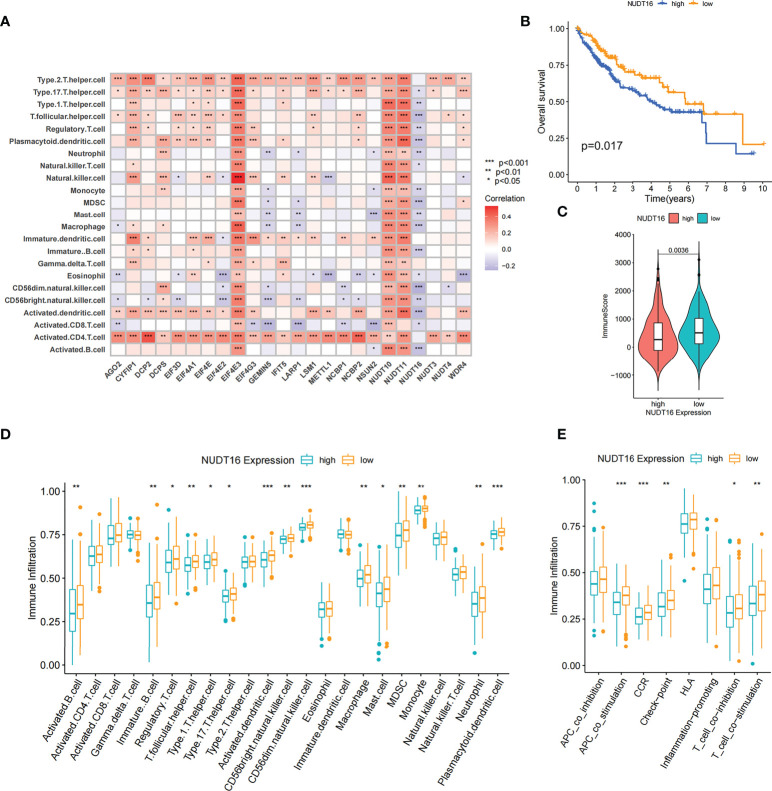
Correlation of TME infiltration with m^7^G regulators and the role of NUDT16 in hepatocellular carcinoma. **(A)** The correlation between each immunocyte and individual m^7^G regulators was analyzed by spearman analysis. **(B)** Overall survival was analyzed using Kaplan-Meier curves for high and low NUDT16 expression subgroups. **(C)** Distribution of immune scores in high and low NUDT16 expression subgroups. **(D)** Comparison of the differences of individual immunocytes between the high and low NUDT16 expressing subgroups. **(E)** Comparison of the differences in immune-related functional levels between high and low NUDT16 expressing subgroups. Asterisks represent statistical p-values (*P < 0. 05; **P < 0. 01; ***P < 0. 001).

By Spearman correlation analysis, the specific correlation among each m^7^G regulator and immune cells infiltration was further characterized (**Figure 5A**). High expression of EIF4E3 and NUDT10/11 remarkably correlated with enhanced immune cell infiltration, whereas expression of *NUDT16* was negatively relevant to the level of immunocyte infiltration. In these m^7^G regulators, we observed a remarkable negative association of NUDT16 with prognosis and immune infiltration (**Figures 5A, B**). We used ESTIMATE algorithm to quantitate the overall immunocyte infiltration in patients with high and low *NUDT16* expression. It was identified that NUDT16 low expression exhibited a higher immune score, implying that NUDT16 low expression enhances immunocyte infiltration in TME, thus corroborating the above results (**Figure 5C**). We then investigated the differences in the specificity of 23 TME-infiltrating immunocytes in patients with high and low *NUDT16* expression. We found significantly increased infiltration of 23 TME immunocytes in the tumor with low *NUDT16* expression compared to those with high expression (**Figure 5D**). We noticed that *NUDT16* was significantly and negatively relevant to the infiltration level of activated dendritic cells and CD8 T cells. Furthermore, we also found that *NUDT16* was seriously and negatively related to the levels of several immune-related functions such as T cell co inhibition, T cell co stimulation, CCR, check point and APC co stimulation (**Figure 5E**).

To further validate the effect of NUDT16 on TME immune infiltration, we performed further analysis using the LIRI-JP cohort. Consistent with previous result, patients with low NUDT16 expression had higher immune score (**Figure S3A**). We further compared the infiltration of aDCs and CD8 T cells between high and low NUDT16 subgroups and showed that they were more infiltrated in the low NUDT16 expression group (**Figures S3B, C**). Based on the above results, we found that NUDT16 was significantly and negatively relevant to the infiltration level of activated dendritic cell, which are responsible for antigen presentation and initial T-cell activation and have a significant role in tumor immunity. To further verify the effect of NUDT16 on the activation of DCs, we examined the expression of specific markers of aDCs in the high and low NUDT16 groups. These markers were identified by Pornpimol et al. and can specifically represent aDCs (37). As expected, the expression of specific markers of aDCs was increased in the low expression group of NUDT16, suggesting that increased expression of NUDT16 may inhibit the activation of DCs (**Figure S3D**). Activation of dendritic cells is dependent on the high expression of MHC molecules, co-stimulatory factors and adhesion factors. We therefore compared the expression of MHC molecules, co-stimulatory factors and adhesion factors in the high and low NUDT16 expression groups. The result showed that CD80, CD86, HLA-DMA, HLA-DMB, HLA-F, HLA-L, ICAM1, ICAM2 and PDCD1 were significantly elevated in the NUDT16 low expression group (**Figure S3E**). This result further demonstrated that high expression of NUDT16 may inhibit the activation of DCs and decrease the expression of MHC molecules, co-stimulatory factors and adhesion factors. Based on these results, we hypothesized that NUDT16 may impede antitumor immune responses by inhibiting the activation of dendritic cells. We also noted that the expression of various inflammatory cytokines such as IL6, IL8, IL10, IL18, CSF1, CSF2, CCL1, CCL2, VEGFA, VEGFB, VEGFC, NGF and FGF1 were increased in the NUDT16 low expression group (**Figure S3F**). Therefore, we also speculated that high expression of NUDT16 may suppress tumor immunity by influencing the expression of multiple immune-related cytokines in TME.

### m^7^G methylation modification patterns in the LIRI-JP cohort

To verify whether the m^7^G correlation type was applicable to other datasets, we performed validation on the LIRI-JP cohort. Similar to the clustering of TCGA-LIHC dataset, unsupervised clustering identified three completely different patterns of m^7^G modifications in LIRI-JP cohort (**Table S4**, **Figures S4A–F**). The three different m^7^G modification patterns differed significantly in the transcriptional profile of m^7^G (**Figure S4F**). m^7^G cluster A was characterized by increased expression of *METTL1, NUDT3/4/11, CYFIP1, EIF4E, EIF4E2, GEMIN5, NCBP2, EIF3D, EIF4A1, LSM1, AGO2*, and decreased expression of NUDT16; m^7^G cluster B had high expression of only *NUDT16*; the expression of *WDR4, DCP2, LARP1* was significantly increased in m^7^G cluster C group (**Figure S4E**). One-way ANOVA also confirmed significant differences in m^7^G regulator expression between the three subtypes (**Figure S4G**). Prognostic analysis also indicated that m^7^G cluster A and B had better survival, while m^7^G cluster C had worse survival (**Figure S4H**).

### DEGs associated with the m^7^G phenotype in hepatocellular carcinoma

**Figure 6:**
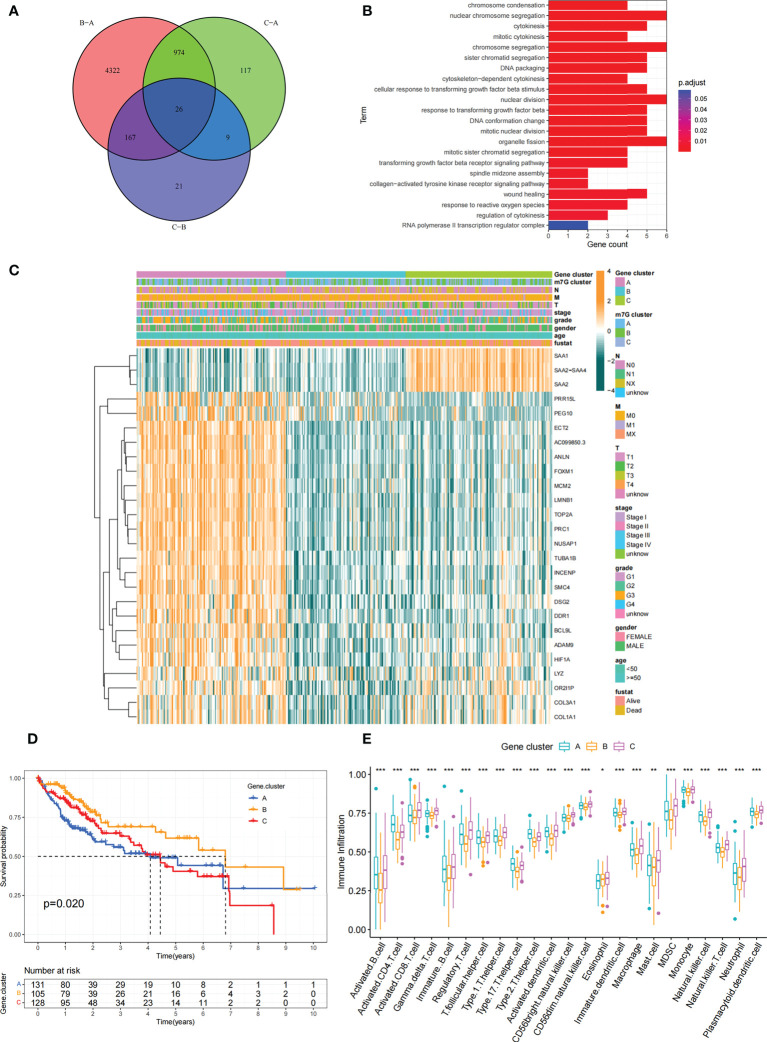
Construction of differential expression of m^7^G gene signatures and functional annotation. **(A)** Venn diagram showing 26 m^7^G-related DEGs between three m^7^G clusters. **(B)** Functional annotation of m^7^G-related genes using GO enrichment analysis. The color depth of the bars represent the amount of gene enrichment. **(C)** Unsupervised clustering of DEGs associated with overlapping m^7^G phenotypes classified patients into different gene clusters, which were called m^7^G gene cluster A, B and **(C)** The m^7^G gene cluster, m^7^G cluster, TMN stage, gender, and age were used as patient annotations. **(D)** Survival curves for m^7^G phenotype-associated genes were plotted. (P=0. 02). **(E)** Abundance of individual TME-infiltrated cells in the three m^7^G gene clusters. Asterisks represent statistical p-values (*P < 0. 05; **P < 0. 01; ***P < 0. 001).

Although we previously classified HCC patients into three different subtypes based on m^7^G regulator expression. However, the genetic alterations between these subtypes remained unclear. Therefore, we further explored the overlapping differentially expressed genes (DEGs) in the distinct m^7^G subtypes. We considered 26 DEGs representing key distinguishing indicators of the three m^7^G modification patterns as m^7^G-associated signatures, which were illustrated with Venn diagrams (**Figure 6A**). GO enrichment analysis of these characteristic genes was conducted and it showed the biological processes associated with RNA modification and transcription were markedly enriched (**Figure 6B**). The results indicated that the overlapping DEGs were characterized by m^7^G modifications and could be considered as m^7^G-associated gene signatures. We performed unsupervised clustering algorithm based on these m^7^G phenotype-associated characteristic genes to obtain three stable transcriptome phenotypes (**Figures S5A–D**). The patients were eventually classified into three distinct subgroups of m^7^G gene signatures with different clinicopathological characteristics, and were redefined as m^7^G gene cluster A-C (**Figure 6C**). These showed that three different m^7^G methylation modification patterns were indeed present in hepatocellular carcinoma. Survival analysis showed statistically remarkable differences in prognosis between three m^7^G gene signatures in HCC samples. m^7^G gene cluster B showed better prognosis for survival, and m^7^G gene cluster A showed worse prognosis (**Figure 6D**). The expression levels of 24 m^7^G regulators in three gene signature subgroups were compared (**Figure S5E**). Consistent with expectations, we could observe remarkable differences in m^7^G regulatory expression among three m^7^G gene clusters. To explore the roles of m^7^G-related phenotypes in immunocyte infiltration, we compared 23 immune cell types among three m^7^G gene clusters (**Figure 6E**). The results showed significant differences in immune infiltration among three m^7^G gene clusters.

### Constructing the m^7^G score and exploring its clinical significance

**Figure 7:**
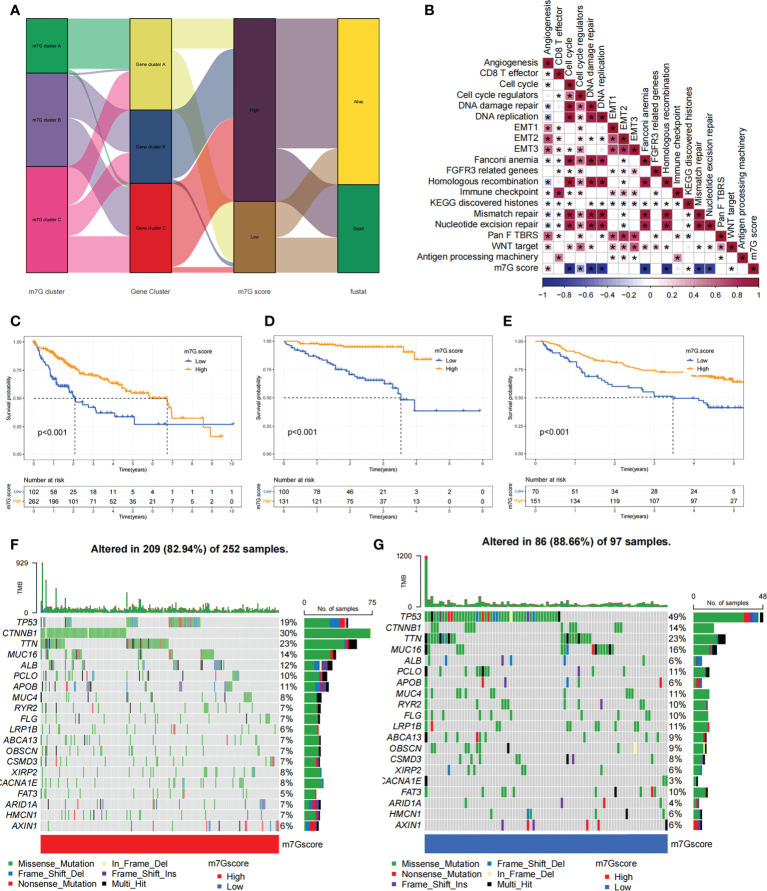
Construction of the m^7^G score and exploration of the relevance of its clinical features. **(A)** Alluvial plots showed changes in m^7^G clusters, gene clusters, m^7^G score and patient survival status. **(B)** Associations between m^7^G scores and certain biogenetic markers were analyzed. Positive associations were colored in red and negative associations were colored in blue. **(C)** Survival curves for the high and low m^7^G score patient subgroups in TCGA-LIHC cohort. P< 0. 001. **(D)** Survival curves for high and low m^7^G score subgroups of patients in LIRI-JP cohort. P< 0. 001. **(E)** Survival curves for high and low m^7^G score subgroups of patients in GSE14520 cohort. P< 0. 001. **(F, G)** The mutation status of SMGs in TCGA-LIHC cohort was divided into high **(F)** and low **(G)** m^7^G score subgroups. Each column represents single patient. The top bar shows the tumor mutation burden(TMB), and the numbers on the right indicates the mutation frequency of individual genes. Asterisks represent statistical p-values (*P < 0. 05).

Our previous studies found roles for m^7^G modification in prognosis and regulation of immunocyte infiltration, but the above discussions were based on patient populations only and were unable to predict accurately m^7^G methylation modification pattern in individual patients. Accordingly, we used previously identified m^7^G-related characteristic genes to construct a system called m^7^G score, which could quantify the pattern of m^7^G modifications in each patient. In consideration of the complexity of m^7^G modification quantification, we used an alluvial diagram (**Figure 7A**; **Table S5**) to illustrate the workflow of m^7^G score construction. These results indicated that m^7^G gene cluster B and C exhibited a higher m^7^G score, whereas m^7^G gene cluster A was linked to a lower m^7^G score (**Figure S6A**). Notably, m^7^G cluster A showed the lowest m^7^G score, which was significantly lower than the other two m^7^G clusters (**Figure S6B**). The relationship between known bio-signatures and m^7^G scores was examined. The heatmap of the correlation matrix showed that the m^7^G score was negatively correlated with DNA damage repair and DNA replication (**Figure 7B**). We further identified the ability of the m^7^G score to predict prognosis in terms of survival outcomes, using a method of dividing patients into subgroups with high or low scores with a critical value of -2.4267. Consistent with expectations, patients with high m^7^G scores in TCGA-LIHC cohort were remarkably relevant to a better prognosis (**Figure 7C**). In addition, a predictive advantage of our established risk model could be seen in the results of the ROC curve analysis (**Figure S6C**). Analysis of multivariate Cox regression models taking into account the gender, age, tumor grade, and TMN stage of patients demonstrated that the m^7^G score was a reliable and independent biomarker for assessing patient prognosis (**Figure S6D**).

We next validated the m^7^G scoring scheme by combining genomic information and clinical features from the LIRI-JP cohort. The m^7^G score was found to have potential prognostic predictive value in the LIRI-JP cohort (**Figure S6E**), and patients with a higher m^7^G score had a significant survival benefit (**Figure 7D**). To further validate the reliability of the m^7^G score, we also used GSE14520 cohort in order to identify the association between the m^7^G score and patient prognosis (**Table S6**). As expected, the high m^7^G score subgroup showed a significant survival advantage relative to the low subgroup (**Figure 7E**). Multifactorial analysis of the GSE14520 cohort similarly supported that the m^7^G score could be considered an independent prognostic factor for HCC (**Figure S6F**). The above findings strongly suggested that m^7^G score could represent the m^7^G modification pattern of HCC patients and predict patient prognosis.

We made further significant mutation gene (SMG) analysis for both high and low m^7^G subgroups in HCC samples. SMG mutation landscape revealed that TP53 (19% *vs*. 49%) had a higher somatic mutation rate in the low m^7^G score subgroup, while *CTNNB1* (30% *vs*. 14%) had a higher somatic mutation rate in the high m^7^G score subgroup (**Figures 7F, G**).

**Figure 8:**
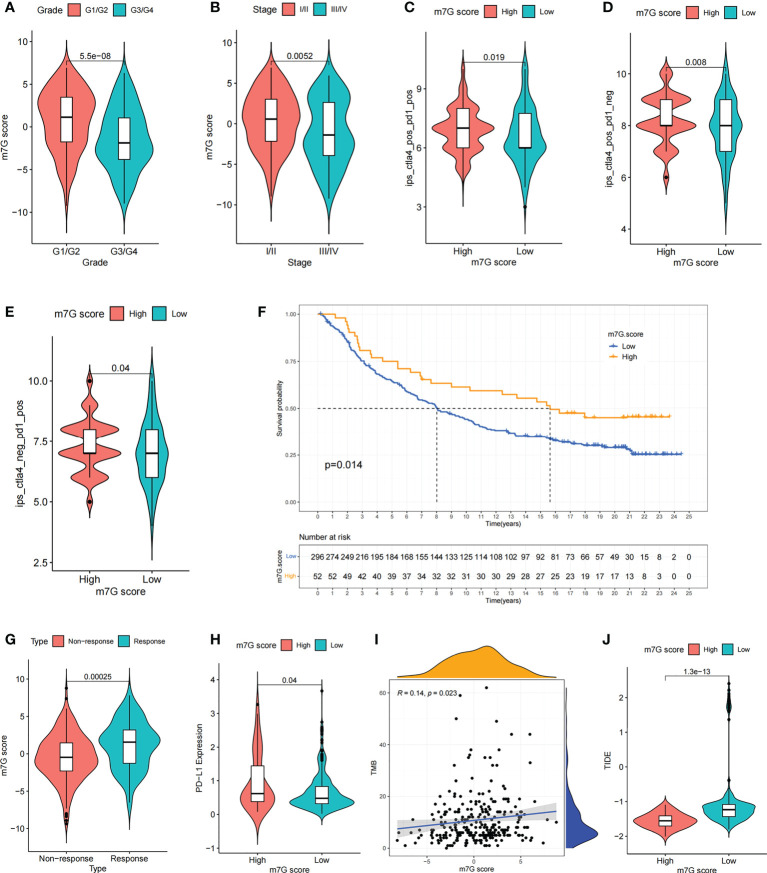
The m^7^G score predicts immunotherapeutic benefits. **(A, B)** Comparison of differences in m^7^G scores between tumor grade subgroups **(A)** and TME stage subgroups **(B)**. **(C–E)** Comparison of differences in IPS scores between high and low m^7^G score subgroups. **(F)** Comparison of m^7^G score between different anti-PD-L1 treatment response subgroups in IMvigor210 cohort. **(G)** Survival curves for high and low m^7^G score subgroups of patients in IMvigor210 cohort. P< 0. 05. **(H)** Comparison of PD-L1 expression between high and low m^7^G score subgroups in IMvigor210 cohort. **(I)** m^7^G score and TMB were significantly and positively correlated in IMvigor210 cohort. **(J)** Comparison of differences in TIDE scores between high and low m^7^G score subgroups in IMvigor210 cohort.

We compared the differences in m^7^G score between distinct clinical trait subgroups and showed that higher tumor grade and higher TME stage were associated with lower m^7^G scores (**Figures 8A, B**). And no significant differences were found in m^7^G scores between age and gender subgroups (**Figures S6G, H**). The above findings suggested that the m^7^G score might be used to assess certain clinical features, like tumor grade and clinical staging.

### The m^7^G score in predicting the efficacy of immunotherapy

Recently, a major breakthrough in antitumor therapeutics has been identified in the form of ICI therapy represented by PD-1/CTLA-4 inhibitors. Besides the well-known TML, MSI, and PD-L1 (45, 46), IPS is strongly recommended for the assessment of immune responses. In our analysis, we found that IPS was remarkably elevated in the high m^7^G scoring group (**Figures 8C–E**). These discoveries indirectly suggested that the characterization of m^7^G modification pattern has an essential role in mediating tumor immune responses.

Given the strong association of m^7^G scores with immune response, the next step was to investigate whether m^7^G-modified signatures could be a predictor of patient response to ICI therapy in an independent immunotherapy cohort. Patients with higher m^7^G score had remarkable benefits of treatment and clinical response to anti-PD-L1 immunotherapy compared to patients with lower m^7^G score in the anti-PD-L1 cohort (IMvigor210) (**Figures 8F, G**). Furthermore, PD-L1 expression was significantly higher in patients with high m^7^G scores (**Figure 8H**). In addition to PD-L1, tumor mutation burden (TMB) and TIDE can also be used to assess the immune response. High TMB is associated with higher treatment response rates and longer survival in patients treated with ICI. Our analysis showed that higher m^7^G score was associated with higher TMB (**Figure 8I**). The TIDE algorithm can be used to assess tumor immune evasion. Higher TIDE score suggests that the tumor is more likely to induce immune evasion, indicating that the tumor is poorer in response to ICI treatment. Our result also showed that TIDE was significantly decreased in the high m^7^G score group, suggesting that patients with high m^7^G score may have a better response to immunotherapy (**Figure 8J**). These results demonstrate that the m^7^G score could potentially be used to predict immunotherapy response in patients. In summary, the results of our study strongly indicate that the m^7^G score could predict patient prognosis and patient response to immunotherapy.

### Comparsion of IC50 of small molecule drugs between different m^7^G clusters

**Figure 9:**
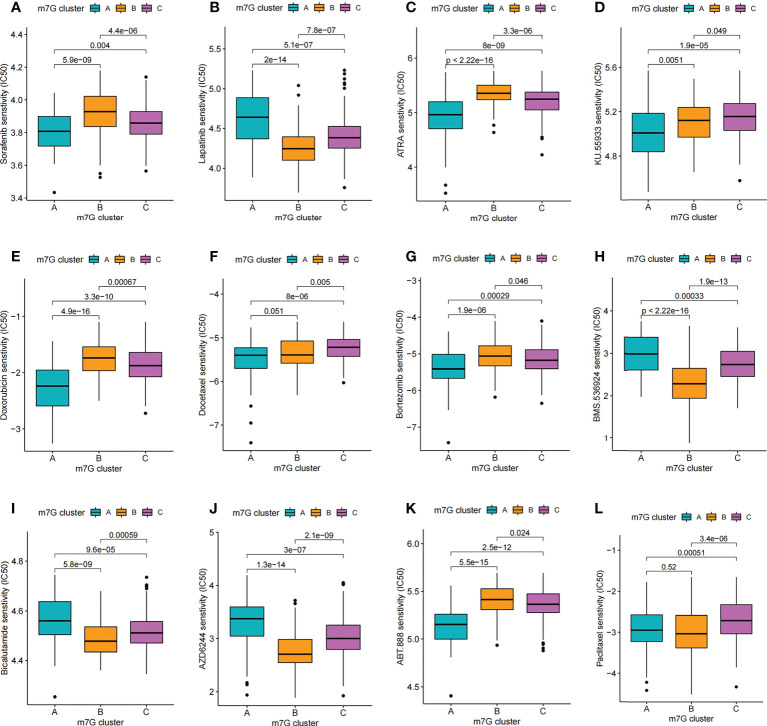
Comparison of drug sensitivity. **(A–L)** Comparison of IC50 of small molecule drugs between different m^7^G clusters.

Through drug sensitivity analysis, 126 small molecule drugs with potential use for the treatment of liver cancer were identified (**Table S7**). Our results indicated that m^7^G cluster A was sensitive to Lapatinib, AZD6244, BMS.536924 and Bicalutamide, while m^7^G cluster B was sensitive to ABT. 888, ATRA, Bortezomib, Doxorubicin and Sorafenib, and m^7^G cluster C was more sensitive to Docetaxel, Paclitaxel and KU.55933 were more sensitive (**Figures 9A–L**). Notably, sorafenib is currently an effective first-line treatment for advanced hepatocellular carcinoma. And our result found that m^7^G cluster B was more sensitive to sorafenib (**Figure 9A**). We then evaluated the relationship between the expression of 24 m^7^G regulators and medication sensitivity (**Table S8**, **Figure S7**). The above results suggest that exploring different m^7^G methylation modification patterns could be used to predict and guide clinical drug therapy for HCC patients.

### Immunohistochemical detection of NUDT16 expression distribution

**Figure 10:**
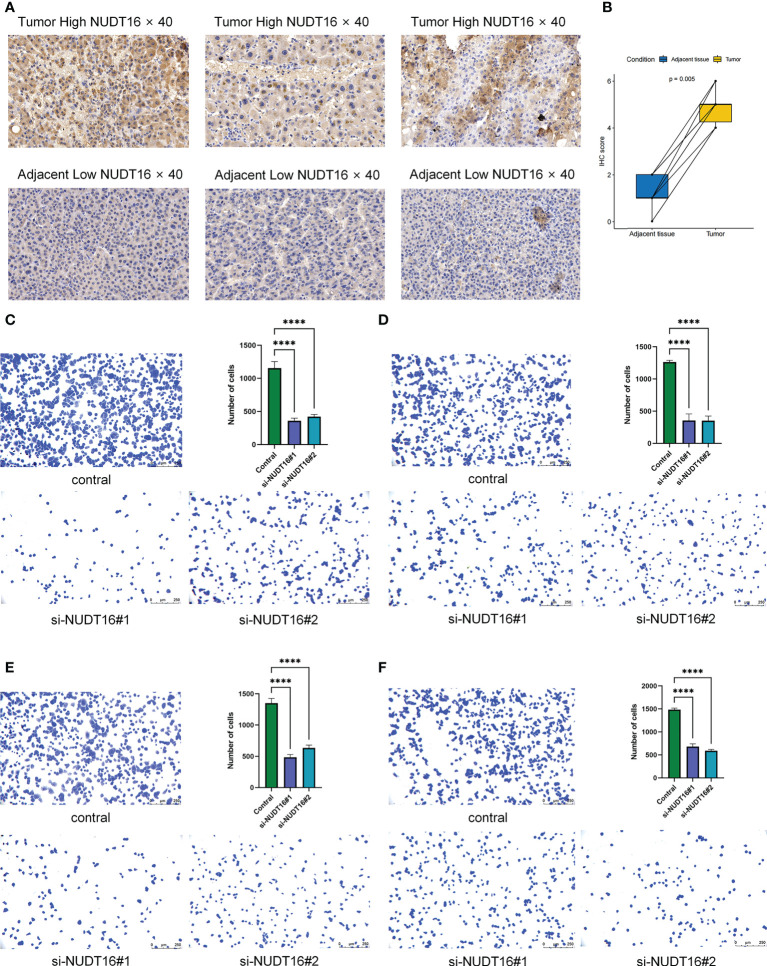
Immunohistochemical analysis of NUDT16 expression in HCC tissues and adjacent tissues and effect of *NUDT16* on the adhesion ability of HCC cells. **(A)** High NUDT16 expression in HCC tissues at 40× magnification. Low NUDT16 expression in adjacent tissues at 40× magnification. **(B)** Comparison of NUDT16 IHC scores in HCC tissues and paired adjacent tissues. **(C)** Comparing the adhesion ability of Huh-7 cells to fibronectin between control and si-NUDT16 groups. **(D)** Comparing the adhesion ability of Huh-7 cells to vitronectin between control and si-NUDT16 groups. **(E)** Comparing the adhesion ability of HepG2 cells to fibronectin between control and si-NUDT16 groups. **(F)** Comparing the adhesion ability of HepG2 cells to vitronectin between control and si-NUDT16 groups. (****P< 0. 0001).

Given the potential role of NUDT16 in hepatocellular carcinoma progression, our previous study comparing the expression of NUDT16 in tumor and normal tissues showed that NUDT16 was highly expressed in tumor tissues. In order to further validate above result, we compared NUDT16 levels in HCC and adjacent non-cancerous tissues. IHC staining indicated that the level of NUDT16 expression in HCC tissues was distinctly higher than that in adjacent non-cancerous tissues (**Figures 10A, B**).

### NUDT16 influenced the adhesion of HCC cells

We previously found that NUDT16 was highly expressed in HCC tissues and that it may influence the immune infiltration in TME. Further, we used cell adhesion assays to explore the effect of NUDT16 on the adhesion of HCC cells to the extracellular matrix. Fibronectin and vitronectin are important component of the extracellular matrix in the TME, so we explored the effect of NUDT16 on the ability of HCC to adhere to fibronectin and vitronectin. The result showed that the adhesion ability of Huh-7 cells to fibronectin (**Figure 10C**) and vitronectin (**Figure 10D**) was decreased in si-NUDT16 compared to the control group. Similarly, we found that the adhesion ability of HepG2 cells to fibronectin (**Figure 10E**) and vitronectin (**Figure 10F**) was decreased in si-NUDT16 compared to the control group.

## Discussion

Liver cancer has a high morbidity and mortality rate, so reliable diagnosis and survival prediction are urgently needed. Increasing evidence suggested that m^7^G modification played integral roles in tumor progression (26, 27). Previous studies on m^7^G methylation modification were mostly focused on individual regulatory molecules such as *METTL1* and *WDR4*, and were less abundant than those on other types of RNA methylation modifications (47–51). The overall features regulated by the combination of multiple m^7^G regulators have not been completely clarified. Even though there are also numerous previous research that has the role of m^7^G regulator-mediated epigenetic regulation in the immune environment (32, 50), there is still little understanding of the overall TME infiltration properties mediated by the combined action of various m^7^G regulators. Therefore, studying the integral features mediated by the combined effects of various m^7^G regulators, and identifying different m^7^G modification patterns in TME will strengthen our understanding of the role of m^7^G methylation in the antitumor immune response and help guide more effective immunotherapeutic strategies.

In this study, we revealed three distinct m^7^G clusters based on 24 m^7^G regulators, and the TME cell permeation characteristics were significantly different among these three subtypes. The m^7^G cluster A was characterized by natural immunity and stromal activation, especially activation of EMT, TGFb signaling pathways, which were thought to be T cell suppressive (43). Thus, the finding that cluster A was abundant in innate immunocyte infiltration but had a poorer prognosis could be explained. m^7^G cluster B and C were characterized by adaptive immune activation, manifested by significant enrichment of antitumor lymphocyte subsets such as T-cell CD8 and NK cells. Taking into account that PD-L1 is a proven biomarker to predict immunotherapy response (44). Our study also found remarkable upregulation of PD-L1 expression in m^7^G A and C subtypes, and this finding may help to predict the efficacy of immunotherapy. Combined with the cell permeation characteristics of TME between the three subtypes, it is quite possible to confirm the reliability of our immunophenotypic classification of the distinct m^7^G clusters. More recent literature has reported that activation of EMT- and TGFb-related pathways could impede lymphocyte infiltration into tumor parenchyma (51), and it has been proposed that targeting specific molecular inhibitors of TGFb could remodel the tumor microenvironment and reinstate antitumor immunity (35, 52). Accordingly, we hypothesize that HCC patients in m^7^G cluster A may benefit from the combination of ICB drugs and TGFb blockade.

In addition, this study found that DEGs with different m^7^G modification patterns were found to be significantly enriched in biological processes involving RNA modification and transcriptional correlates in this study, and these DEGs were recognized as m^7^G-related signature genes. We identified three gene clusters based on m^7^G signature genes and found that they clustered similarly with m^7^G modifications, and both were significantly correlated with patient survival prognosis. In order to quantitate the pattern of m^7^G modification in individual HCC patients and thus more precisely guide the treatment strategy for individual patients, we further developed “m^7^G score”, a quantitative system to define it. According to this scoring system, m^7^G subtypes B and C, which were characterized by adaptive immune activation, presented a higher m^7^G score. In contrast, the m^7^G A subtype, characterized by natural immunity and significant stromal activation, presented a lower score.

What’s more, we found that the m^7^G score was significantly relevant with IPS, a predictor of immune response. This finding implies that our m^7^G score has a potential predictive advantage in the precise immunotherapy of liver cancer. In fact, we validated the constructed m^7^G scoring system with three liver cancer cohorts (TCGA-LIHC cohort, LIRI-JP cohort, GSE14520 cohort) and confirmed our hypothesis that the m7G score would be regarded as an independent prognostic biomarker of HCC. Moreover, we validated that the m^7^G score is a reliable predictor of patient survival outcomes and immunotherapy response through another independent ICI cohort (IMvigor210).

While elucidating the results of m^7^G modification clustering, we also discussed the roles of individual m^7^G regulators specifically in tumor immunomodulation. Dai et al. found in their recent studies that *METTL1* and *WDR4* were upregulated in patients with intrahepatic cholangiocarcinoma(ICC) and that METTL1-mediated m^7^G tRNA modification selectively regulated oncogenic genes including EGFR pathway and cell cycle genes in ICC through a codon frequency-dependent mechanism mRNA translation. This is associated with poor prognosis in ICC patients (53). Meanwhile, numerous studies have also disclosed the functions and potential mechanisms played by *METTL1* and *WDR4* in other cancer types, as they were found to significantly upregulate and regulate the translation of oncogenic mRNAs in multiple cancer types, and they were considered to be a tumorigenic oncogene (54, 55). In our research we also found that *METTL1* and *WDR4* expression was upregulated in hepatocellular carcinoma tissues and correlated with reduced survival time, and also found that high *METTL1* and *WDR4* expression was distinctively and negatively relevant with the level of NK cell infiltration. In addition, *NUDT16* was brought to our attention in our study because of its remarkable negative association with both tumor prognosis and immunocyte infiltration. Thereafter, we focused on analyzing the specific association between *NUDT16* and immunocyte infiltration, while comparing the expression of *NUDT16* in HCC tissues and adjacent non-cancerous tissues by IHC staining. Based on these findings, we hypothesized that *NUDT16*-mediated m^7^G methylation modification might impede antitumor immune responses by inhibiting the activation of DCs. In this study, *NUDT16* was also found to be commonly altered and significantly upregulated in CNV among tumor tissues, indicating that it may also promote the development and progression of HCC. In the future, we need to further validate the effects of *NUDT16*-mediated m^7^G modification on tumor immunosuppression mechanisms in biological experiments such as cell culture and mouse models.

The assessment of human tumor significant mutation gene (SMG) is an important foundation for cancer diagnosis, treatment and rational choice of therapy. Previous studies have shown that TP53 mutations occur in many tumor types and suppress antitumor immune responses (56). In our study, the somatic mutation rate of TP53 was found to be significantly higher in the low m^7^G score subgroup of m^7^G than in the high m^7^G score subgroup. These tumor driver gene mutations associated with m^7^G scores were significantly associated with tumor immunity, showing that there is a complex interaction between m^7^G modifications and tumor immunogenomic features.

Nevertheless, our study is not without any limitations. Although our model has incorporated 24 recognized m^7^G RNA methylation regulators, more need to be incorporated in the future to optimize our model and improve the accuracy of prediction. Although we have performed multiple validations of the resulting prediction models, more independent datasets could be incorporated in the future to reduce potential biases. On the other hand, as the main findings of this study were obtained through comprehensive bioinformatics analysis, biological experiments such as cell culture and mouse models are needed to further explore the detailed mechanisms of how m^7^G regulators interplay with each other.

In this study, we performed a comprehensive evaluation of m^7^G modification patterns in 816 hepatocellular carcinoma samples based on 24 m^7^G regulatory factors and made a systematic correlation of these modification patterns with TME cells infiltration characteristics. These comprehensive analyses revealed a broad regulatory mechanism of m^7^G methylation modifications on tumor microenvironment. In short, differences in m^7^G modification patterns are a non-negligible factor contributing to the heterogeneity and complexity of the individual tumor microenvironment. Assessing the m^7^G modification patterns of individual tumors would help enhance our perception of TME infiltration characteristics and provide significant insights into immunotherapy efficacy.

## Data availability statement

The original contributions presented in the study are included in the article/**Supplementary Material**. Further inquiries can be directed to the corresponding author.

## Ethics statement

The studies involving human participants were reviewed and approved by Ethics Committee of Beijing Youan Hospital. The patients/participants provided their written informed consent to participate in this study.

## Author contributions

LW contributed to the design, writing, experiments and data analysis of the manuscript. FM reviewed and edited the manuscript. FM, XiL and YF recruited patients and obtained samples. All authors contributed to the article and approved the submitted version.

## Funding

This work was supported by the National Science and Technology Major Project (2018ZX10302205-005).

## Conflict of interest

The authors declare that the research was conducted in the absence of any commercial or financial relationships that could be construed as a potential conflict of interest.

## Publisher’s note

All claims expressed in this article are solely those of the authors and do not necessarily represent those of their affiliated organizations, or those of the publisher, the editors and the reviewers. Any product that may be evaluated in this article, or claim that may be made by its manufacturer, is not guaranteed or endorsed by the publisher.

## Abbreviations

AUC, Area under the curve; DEGs, Differentially expressed genes; EMT, Epithelial-mesenchymal transition; HCC, Hepatocellular carcinoma; ICC, Intrahepatic cholangiocarcinoma; ICI, Immune checkpoint inhibitor; IHC, Immunohistochemical; m^7^G, N^7^-methylguanosine; PCA, Principal component analysis; ROC, Receiver operating characteristic; SMG, Significant mutation gene; TME, Tumor microenvironment.
