# Supplementary material for: m7G regulator-mediated methylation modification patterns define immune cell infiltration and patient survival
Source: Front Immunol. 2022 Oct 28;13:1022720. doi: 10.3389/fimmu.2022.1022720 (PMC9650241; doi:10.3389/fimmu.2022.1022720)
Supplement: Supplementary file 1 [file DataSheet_1.pdf]

Supplementary Material

Supplementary Figures

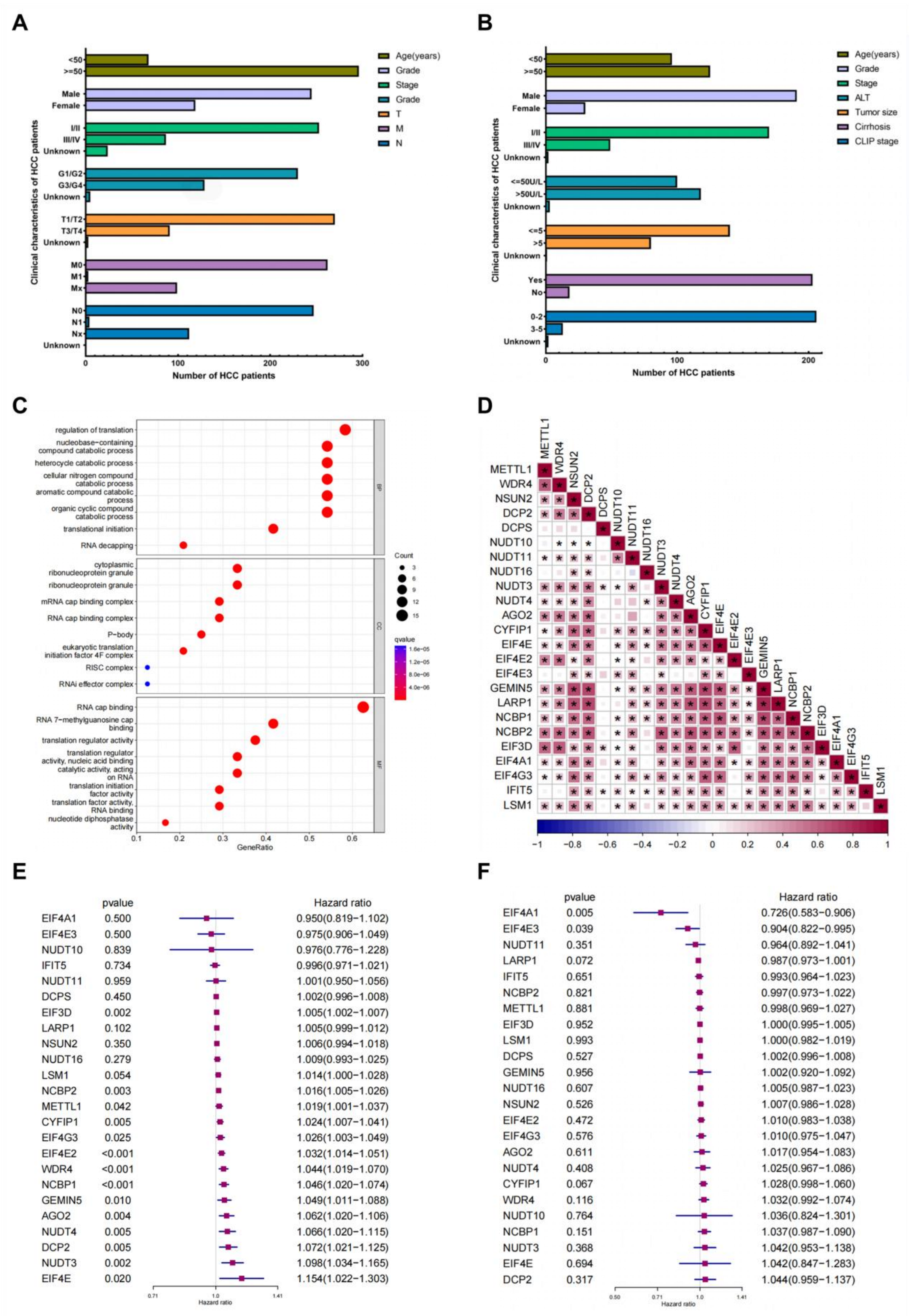

Supplementary Figure 1. Clinical characteristics of HCC patients and correlation and prognostic analysis of 24 m^{7}G regulators. (A) Clinical characteristics of HCC patients in TCGA-LIHC cohort. (B) Clinical characteristics of HCC patients in GSE14520 cohort. (C) GO enrichment analysis of the 24 m^{7}G regulators. The x-axis indicated the number of genes enriched. (D) Correlations between these m^{7}G regulators were calculated in HCC using Spearman correlation analysis. Negative correlation: blue; Positive correlation: purple. (*P<0.05). (E-F) The prognostic analyses for the 24 m^{7}G regulators by univariate Cox regression (E) and multivariate Cox regression models (F).

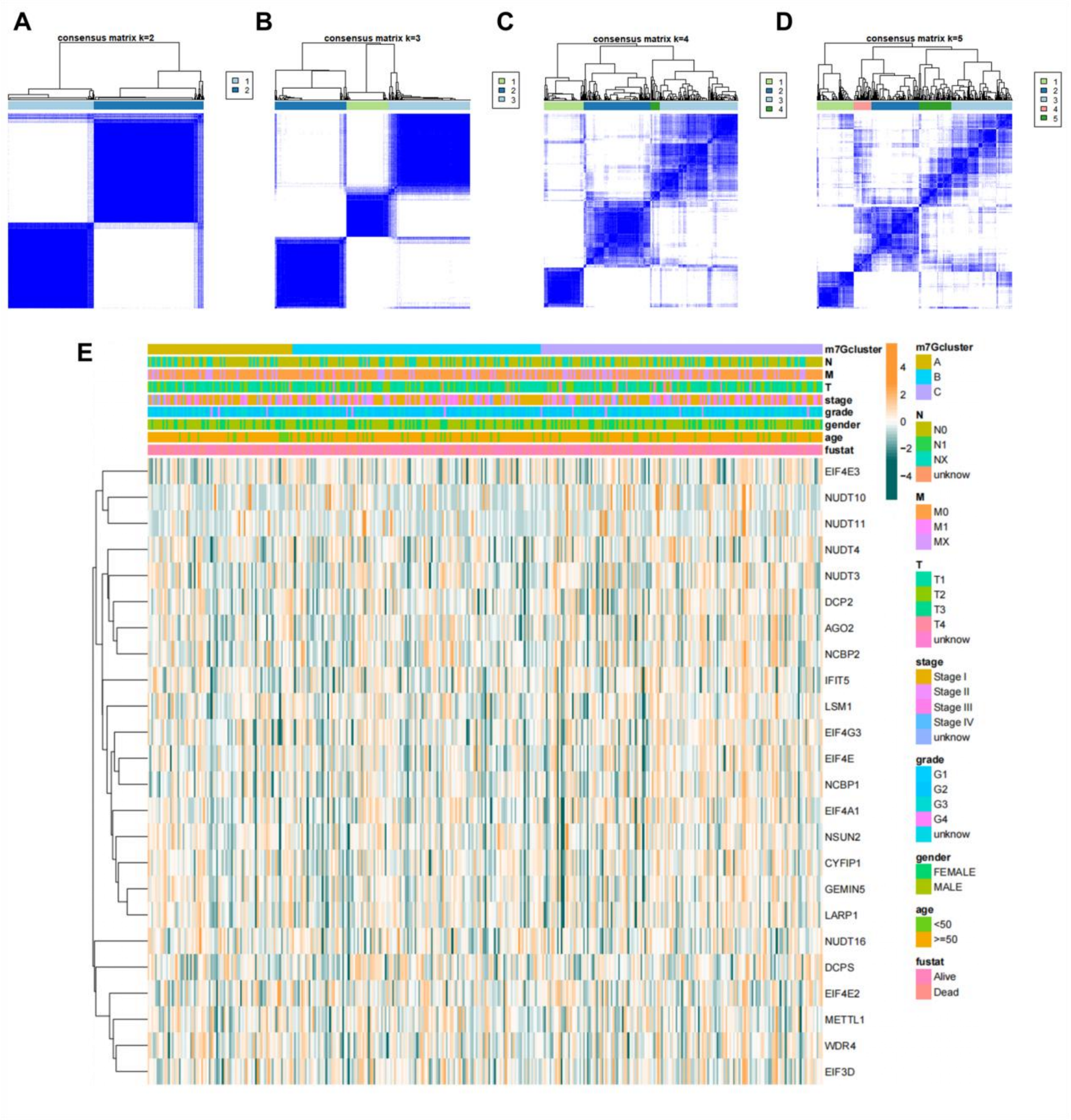

Supplementary Figure 2. Unsupervised clustering of 24 m^{7}G regulators in TCGA-LIHC cohort. (A-D) Consensus matrices of TCGA-LIHC cohort for k = 2 - 5. (E) Unsupervised clustering of 24 m^{7}G regulators in TCGA-LIHC cohort. The m^{7}G-clusters and cohort names were used as patient annotations. Each column represented patients and each row represented m^{7}G regulators. Clinicopathological information including age, gender, tumor stage, and m^{7}G cluster is shown in the

annotation above. Yellow represented high expression of regulators and green represented low expression.

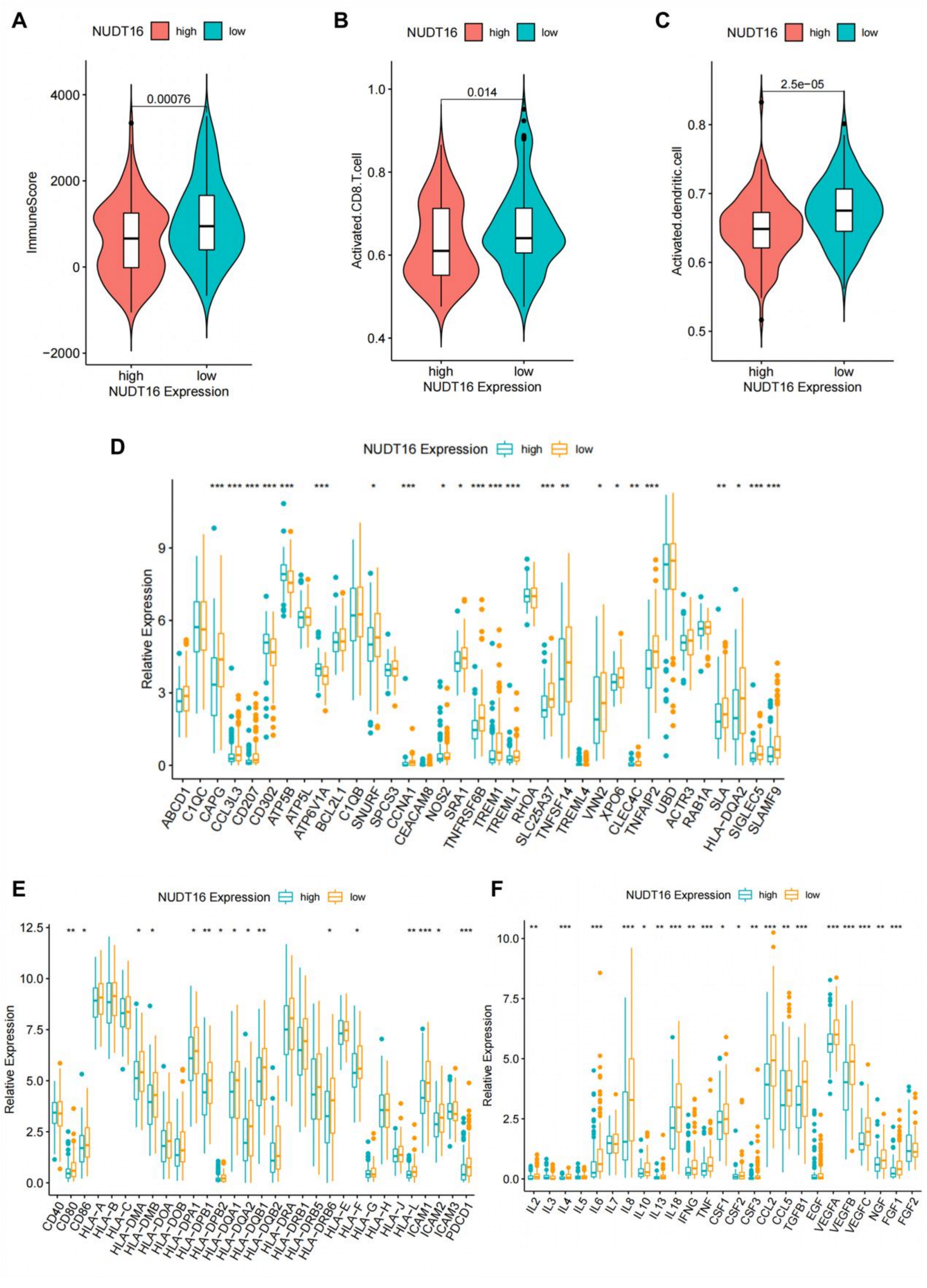

Supplementary Figure 3 The role of NUDT16 in HCC tumor immunity. (A) Distribution of immune scores in high and low NUDT16 expression subgroups. (B) Comparison of the differences of aDCs between the high and low NUDT16 expressing subgroups. (C) Comparison of the differences of CD8 T cells between the

high and low NUDT16 expressing subgroups. (D) Comparing the expression of specific markers of aDCs between the high and low NUDT16 expressing subgroups. (E) Comparing the expression of MHC molecules, co-stimulatory factors and adhesion factors between the high and low NUDT16 expressing subgroups. (F) Comparing the expression of inflammatory cytokines between the high and low NUDT16 expressing subgroups.

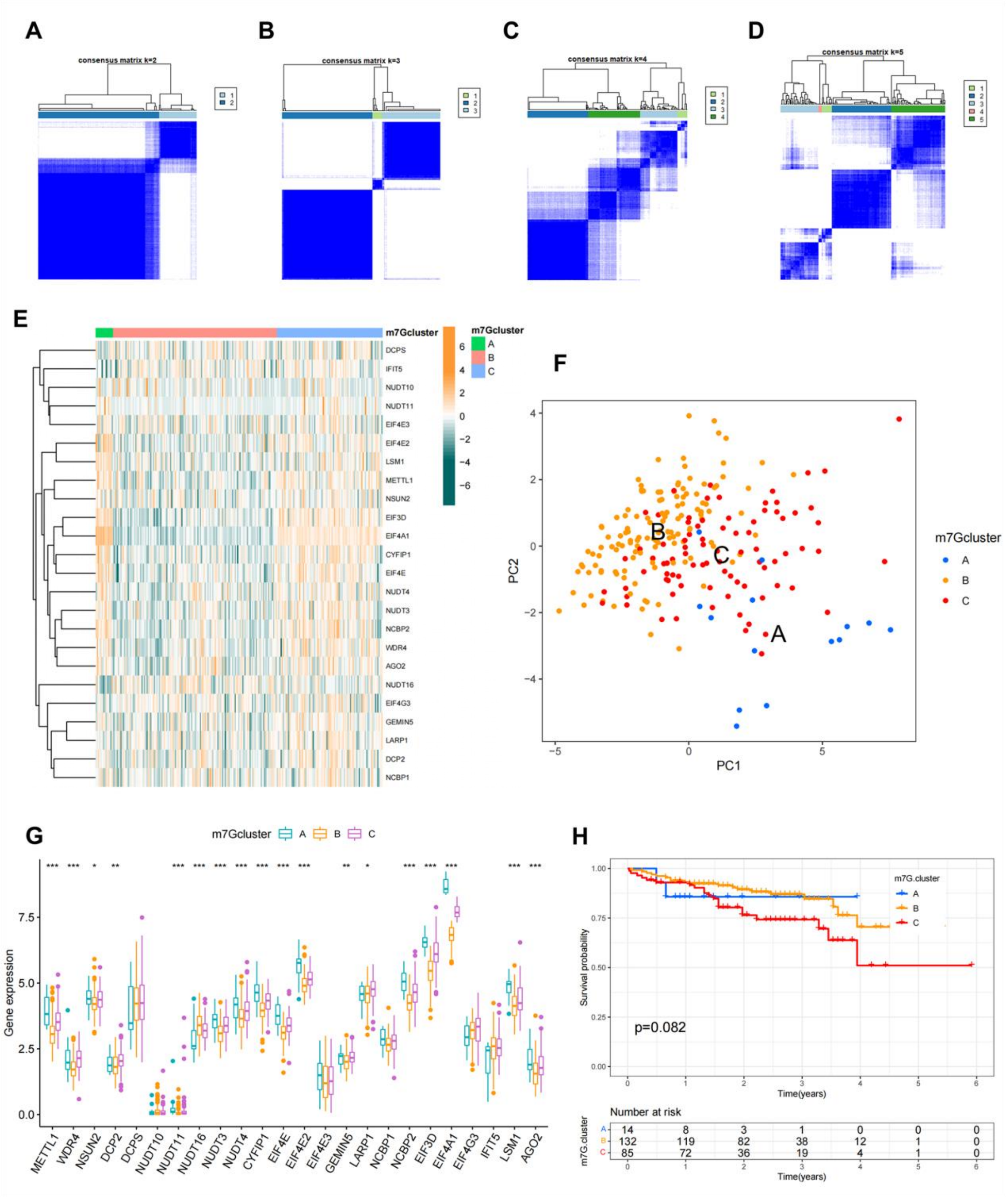

Supplementary Figure 4. Unsupervised clustering of 24 m^{7}G regulators in LIRI-JP cohort. (A-D) Consensus matrices of LIRI-JP cohort for k = 2 - 5. (E) Unsupervised clustering of 24 m^{7}G regulators in LIRI-JP cohort. Yellow represented high expression of regulators and blue represented low expression. (F) PCA of the transcriptome profiles of the three m^{7}G clusters revealed remarkable differences

between the transcriptomes of the different clusters. (G) The expression of 24 m^7G regulators in the three m^7G clusters. Lines in the boxes indicated median values and scatters indicated outliers. The upper and lower ends of the boxes indicated the interquartile range of values. Asterisks indicated statistical p-values(*P < 0.05; **P < 0.01; ***P < 0.001). (H) Survival analysis for three m^7G clusters in LIRI-JP cohort, including 14 samples in m^7G cluster A, 132 samples in m^7G cluster B and 85 samples in m^7G cluster C. It can be tentatively speculated that the overall survival of m^7G cluster C was worse than the other two (p=0.082).

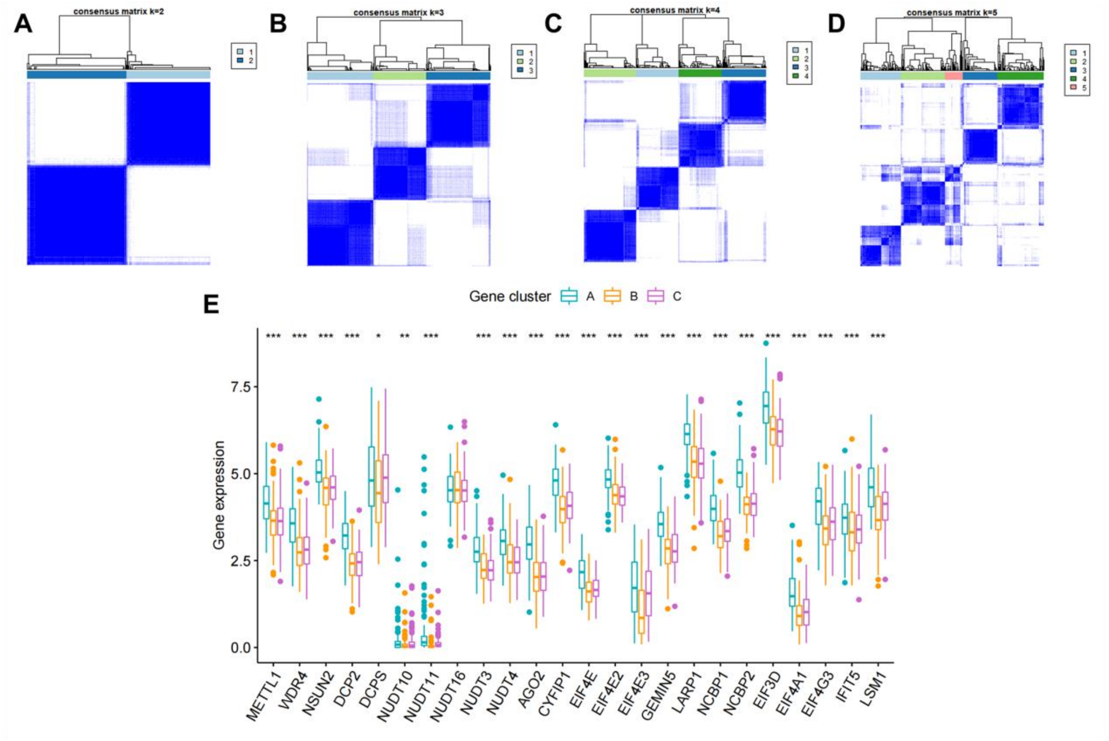

Supplementary Figure 5. Unsupervised clustering representation of 26 m^7G phenotype-associated genes in the liver cancer cohort. (A-D) Consensus matrices of TCGA-LIHC cohort for k = 2 - 5. (E) The expression of 24 m^7G regulators in three gene signature subtypes.

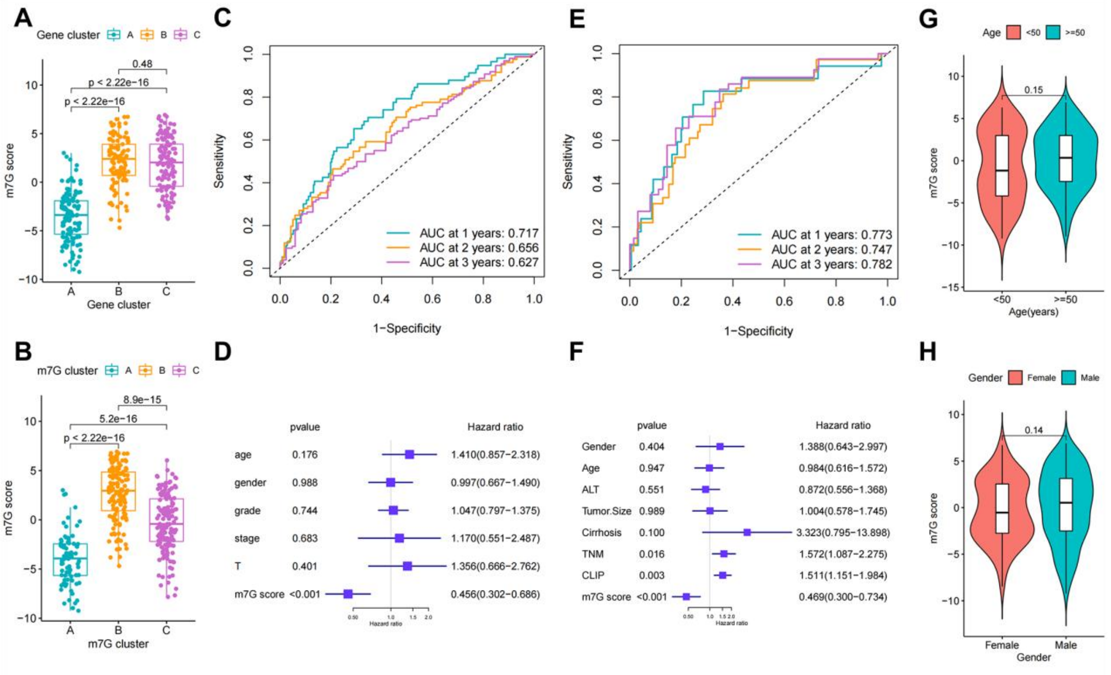

Supplementary Figure 6. The m^{7}G score associated with survival-related outcomes. (A) Differences in m^{7}G scores between the three m^{7}G gene clusters in the TCGA-LIHC cohort (P<0.001). (B) Differences in m^{7}G scores between the three m^{7}G clusters in TCGA-LIHC cohort (P<0.001). (C) Predictive value of m^{7}G score measured by ROC curve in TCGA-LIHC cohort. (D) Multifactorial Cox regression subgroup analysis was performed to assess the clinical prognostic value of the m^{7}G score in the TCGA-LIHC cohort. The length of the horizontal line represents the 95% confidence interval for each group. (E) Predicted values of m^{7}G scores in the the LIRI-JP cohort measured by ROC curves. (F) Multifactorial Cox regression subgroup analysis was performed to assess the clinical prognostic value of the m^{7}G score in the GSE14520 cohort. (G-H) Comparison of differences in m^{7}G scores between age subgroups(G) and gender subgroups(H).

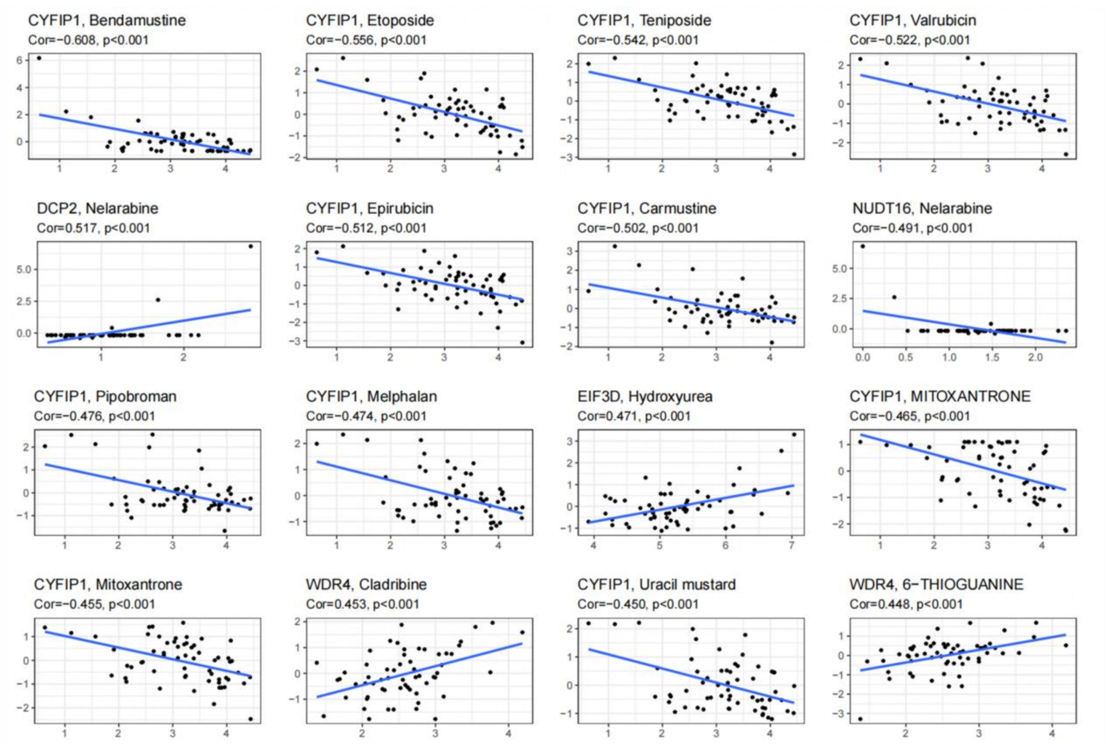

Supplementary Figure 7. Correlation of 24 m^{7}G regulators expression levels and IC50 of different drugs.
